# Liver Machine Perfusion—When Physiopathology Matters

**DOI:** 10.3390/jcm11175124

**Published:** 2022-08-31

**Authors:** Ruelan Furtado, Laurence Weinberg, Marcos Vinicius Perini

**Affiliations:** 1 Department of Surgery at Austin Health, The University of Melbourne, Melbourne 3010, Australia; 2 Department of Critical Care, The University of Melbourne, Melbourne 3010, Australia

Liver transplantation (LT) has become a victim of its own success. Advances in the field of LT have led to superior outcomes, and patients with diseases such as hilar cholangiocarcinoma and colorectal liver metastases, which were once considered as contraindications for surgery, are now eligible for transplantation. This expanded eligibility has forced LT practitioners to increase the size of the donor pool by expanding the acceptance criteria (allowing for organs from marginal donors to be transplanted) and investigating alternative solutions to rescue organs that would otherwise be discarded because they carry a high risk of post-transplant failure.

Attempts to widen the pool of donors in order to meet demand has yielded tissue from older, fatty livers and comorbid donors and led to donation after circulatory death (DCD), in addition to tissue retrieved from traditional dead-brainstem donors (DBD). Grafts from these extended criteria donors (ECD) have higher rates of primary nonfunction, early graft dysfunction, and the formation of non-anastomotic biliary strictures when transplanted. These postsurgical side effects worsen patient outcomes and increase healthcare costs. Additionally, transplant surgeons are less likely to use ECD grafts after inspecting the quality of the tissue.

Given the current trends in aging, obesity and diabetes, it is expected that the annual utilisation rate of liver grafts in the US will fall from a peak of 78% in 2010 to 44% by 2030, thus decreasing the number of LTs performed [1].This is despite a current decrease in the LT waitlists to 15% in the US and 12% in the UK, attributable to patient death or deterioration. Machine perfusion (MP) of retrieved organs can reverse this trend by reducing the impact of, and measuring the tissue response to, ischaemia-reperfusion injury. The term MP describes several techniques that maintain cellular respiration rather than suppressing it.

The concept of MP is not new. Thomas Starzl and his team used the technique upon the inception of LT procedures. Brettschnider first described the outcomes of experiments using MP for canine livers. The researchers performed dual perfusion of the portal vein and hepatic artery with heating and hyperbaric oxygenation and compared these tissues to cold perfused livers. While the cold perfused livers required immediate transplantation, they found that the livers treated with MP could be sustained for 8 to 24 h before transplantation [2]. The following year, Starzl described the same technique in an early report on LT procedures, which were performed on eight humans. Despite this success, a more economical solution supplanted MP: the flushing of the donor tissue with cold preservation solutions and the storage of the graft in static cold storage (SCS)—essentially, a box of ice.

While SCS reduces the graft metabolism by 10–12 times, the anaerobic activity continues, leading to the generation of oxygen free radicals and, ultimately, ischaemia-reperfusion injury when the clamps are removed. The basic principle of MP is that cellular respiration can replace cold ischaemia. In practice, there are several methods used to achieve this. Two common methods are hypothermic MP (HMP) and normothermic MP (NMP).

In HMP, the cellular metabolism and ATP demand are low. The HMP grafts are connected to a pump that supplies the portal vein with preservation fluid, similar to the Belzer solution—a commonly used cold perfusion fluid. The mechanism of action is considered to work by removing metabolic waste products and protecting the endothelium. Building on this technique, oxygen can be added to the portal perfusion circuit in order to perform hypothermic oxygenated machine perfusion (HOPE). Additionally, oxygen may be added to the hepatic arterial circulation in order to provide a dual oxygenated circuit (D-HOPE), in which the portal vein and hepatic artery are perfused with oxygenated fluid. Experimental results have shown that using oxygenated preservation fluid reduces the accumulation of succinate in the mitochondria, which reduces the production of oxygen free radicals and the likelihood of ischaemia-reperfusion injury.

A randomised, multicentre trial investigating D-HOPE vs. SCS after DCD demonstrated a decreased incidence of intraoperative postreperfusion syndrome (i.e., intraoperative haemodynamic changes), graft dysfunction, and biliary complications in patients who received grafts perfused using the D-HOPE protocol. Furthermore, four times fewer post-transplant biliary interventions were reported in the D-HOPE group compared to the SCS group [3].

While the previously mentioned trial only assessed DCD livers, HMP can also be used for ECD-DBD livers. A multicentre, randomised trial investigating HOPE vs. SCS in the latter group of donors reported a reduction in the postoperative peak ALT levels, complications at the 90-day postoperative mark, and the length of hospital stay for patients who received livers treated with HOPE. In contrast to the trial examining the effects after DCD, the ECD-DBD recipients did not have significantly lower rates of graft dysfunction or biliary complications [4]. In summary, HMP could lead to improved intraoperative outcomes, fewer long-term costs, and an improved patient quality of life.

There are a few differences between HMP and NMP. As the hepatocyte metabolism in the graft is suppressed during MP, there are two consequences. First, HMP has a smaller theoretical risk of graft loss than NMP. Additionally, if there is a failure in MP and the perfusion ceases, a graft connected to HMP will not be subjected to warm ischaemia. Furthermore, keeping the graft in hypothermic conditions allows for conventional retrieval, initial flush, and transport in SCS and does not require the transportation of specialised equipment by the retrieval team.

In contrast, NMP preserves the liver in near-physiological conditions. The system uses a blood-based perfusate that maintains the cellular metabolism and experimentally inhibits inflammation and prevents cell death. Previous experiments assessing gene expression in NMP grafts identified significant activity in terms of growth, metabolism, and tissue repair [5]. The perfusion of the graft also allows for reliable viability testing while it is connected to MP. As a result, marginal grafts from the ECD pool are more likely to be used, as transplant surgeons have access to objective data on the hepatocyte function. This paradigm is also now being extended to HMP.

In one retrospective study, 31 ‘orphan’ ECD grafts, which were refused by all LT centres in a UNOS region, were accepted and placed on HMP after SCS. Viability testing was conducted by sampling the HMP effluent prior to transplantation. Comparing the patient outcomes with a historical matched cohort led to the identification of improved outcomes for HMP patients in terms of early graft dysfunction, the hospital length of stay, and biliary complications at 1-year post-transplantation. This trial demonstrated that ECD grafts can be safely tested and transplanted, that HMP can overcome the logistical problems of transferring organs between regions, and that the grafts can be tested for viability [6].

The situation of NMP is more complicated than that of HMP because there are inconsistencies in the application of NMP. Some protocols minimise the cold ischaemia time by initiating perfusion with a transportable MP at the retrieval hospital, and others initiate MP at the transplant centre after the SCS and transport. Both methods have been associated with the increased use of ECD grafts and superior outcomes compared to SCS [7,8].

One multicentre, randomised trial compared NMP to SCS in adult DBD and DCD patients. Despite identifying longer functional warm ischaemia and overall storage times in the NMP DCD grafts, the authors reported better outcomes with the use of NMP. Indeed, NMP DCD grafts had better rates of early allograft dysfunction and peak AST levels compared to the SCS DBD grafts. However, the rates of biliary complications in this study were not significantly different, as both DBD and DCD grafts were included [8].

While these studies demonstrated the utility of MP, they raise questions as to which type of MP is most effective, given the circumstances of the donor, the recipient, and available logistics. Another conclusion that can be drawn is that, compared to SCS, MP has shown no benefits for patient or graft survival in any of the trials mentioned above. This might be because primary nonfunction and mortality are relatively uncommon events compared to early graft dysfunction.

A less widely published technique of MP is to perfuse the organs before they are removed from the donor. Normothermic regional perfusion (NRP) involves placing cannulae in situ, before or during tissue retrieval. Normothermic perfusion seems to be the ideal technique for collecting tissue from DCD. The withdrawing of life support in this group is associated with physiological changes in peripheral and mesenteric vasoconstriction caused by catecholamine release upon brainstem injury. This occurs before the declaration of death and is compounded by warm ischaemia prior to cross-clamping. Thus, even before circulatory arrest and the declaration of death, grafts can become ischaemic, which cannot be stopped by cold perfusion. In NRP, oxygenated blood perfuses the abdominal organs via an extracorporeal circuit consisting of an oxygenator, heater, and pump. The circuit is completed soon after the declaration of death and continues running for 2 h, after which a cold perfusion fluid is run through the circuit. A randomised trial found significantly lower rates of early graft dysfunction (12% vs. 32%), no instances of cholangiopathy (0% vs. 27%), and a doubling of the organ utilisation rate (61% vs. 27–36%) for NRP compared to standard DCD, respectively. In France, Italy, and Norway, abdominal NRP is the standard procurement procedure for DCD [9]. It is not clear whether the economic data on cost-effective health support this. This procedure requires a perfusionist to attend the retrieval hospital with a machine and perform 4–6 h of in situ perfusion. However, as multiple organs are perfused with NRP, this process may benefit multiple recipients. Additionally, there are lower costs associated with the cannulation equipment used for this technique.

However, using NRP raises the ethical issue of when the cannulae should be inserted into the donors. While the premortem placement of endovascular occlusive balloons and perfusion cannulae is beneficial for the grafts, initiating the retrieval process before death might be unacceptable for some patient groups. However, in the study by Watson et al., the reported benefits were observed following the *post-mortem* cannulae placement [9].

A common variable in all these studies is the effect that MP has on logistics. In most cases, the effect is beneficial, as time on MP often means that LT can be delayed until daytime, which is generally considered a safer time to operate. In other ways, MP is cumbersome, as perfusion devices and technicians are required to monitor the equipment and may be required at the retrieval hospital. This requirement increases upfront costs; however, this should be mitigated by downstream cost savings from reduced morbidity. Finally, MP has been shown to increase the utilisation of grafts in ECD. Thus, the appeal of MP has come full circle, to the point when Brettscheider first demonstrated its efficacy.

In conclusion, several systems exist for performing MP using LT grafts. Although the techniques vary in terms of their mechanisms and applications, thus far, all techniques have shown benefits for LTs from ECD-DBD and DCD donors. Determining which method is appropriate in specific circumstances is the next challenge for researchers. Each MP system has benefits, and it might be the case that HMP and NMP are complementary techniques. The results of upcoming multicentre trials, expected in the next 2 years, should elucidate these differences.
